# Role of intraoperative feeding jejunostomy in esophageal cancer surgery

**DOI:** 10.1186/s13019-022-01944-1

**Published:** 2022-08-20

**Authors:** Min Soo Kim, Sumin Shin, Hong Kwan Kim, Yong Soo Choi, Jae Il Zo, Young Mog Shim, Jong Ho Cho

**Affiliations:** Department of Thoracic and Cardiovascular Surgery, Samsung Medical Center, Sungkyunkwan University School of Medicine, 81 Irwon-ro, Gangnam-gu, Seoul, 06531 South Korea

**Keywords:** Feeding jejunostomy, Ivor Lewis operation, Esophagectomy, Complications

## Abstract

**Background:**

Feeding jejunostomy was routinely placed during esophagectomy to ensure postoperative enteral feeding. Improved anastomosis technique and early oral feeding strategy after esophagectomy has led to question the need for the routine placement of feeding jejunostomy. The aim of this study is to evaluate role of feeding jejunostomy during Ivor Lewis operation.

**Methods:**

We retrospectively reviewed 414 patients who underwent the Ivor Lewis operations from January 2015 to December 2018.

**Results:**

61 patients (14.7%) received jejunostomy insertion. The most common indication for jejunostomy was neoadjuvant concurrent chemoradiation therapy (CCRT). 48 patients (79%) had jejunostomy removed within 60 days after the surgery and the longest duration of jejunostomy inserted state was 121 days. About two-third of the patients with jejunostomy had never prescribed with an enteral feeding product. Among 353 patients without intraoperative feeding jejunostomy, 11(3.1%) received delayed jejunostomy insertion. Graft-related problems (6 patients), cancer progression (3 patients), acute lung injury (1 patient), and swallowing difficulty (1 patient) were reasons for delayed feeding jejunostomy insertion. Complication rate was relatively high as 24 patients (33.3%) out of 72 patients with jejunostomy insertion had complications and 7 patients (9.7%) visited ER more than twice with jejunostomy-related complications.

**Conclusion:**

Only 3.6% patients who underwent the Ivor Lewis operation during 4-year span had anastomosis leakage. Although one-third of the patients with jejunostomy were benefited with alternative method of feeding after discharge, high complication rate regarding jejunostomy should be also considered. We believe feeding jejunostomy should not be applied routinely with prophylactic measures and should be reserved to very carefully selected patients with multiple high-risk factors.

## Introduction

Esophagectomy is a high-risk procedure associated with numerous morbidities and relatively high mortality, with an incidence of complications associated with the surgery between 17 and 74% [1]. The use of feeding jejunostomy has been a routine procedure for esophageal cancer surgery to ensure enteral feeding after esophagectomy. The benefits of early enteral feeding after esophagectomy have been well-recognized with improved immunity, preserved gut integrity, and reduced complications [2, 3]. Feeding jejunostomy may serve as insurance for anastomosis leakage or an alternative feeding route for patients with poor oral intake [4]. However, the incidence of anastomosis leakage and mortality has continuously declined with standardization of surgical procedures, advancement in instruments, and improved postoperative care [5]. Furthermore, the trend toward early oral feeding after gastroenteric surgery makes surgeons wonder if feeding jejunostomy is necessary after the Ivor Lewis operation because the quality of life after esophagectomy is now considered an important treatment aspect for esophageal cancer [6]. The trend of omitting feeding jejunostomy after the Ivor Lewis operation may exist because many believe that feeding jejunostomy brings unnecessary complications and lowers the quality of life. In addition, a recent study reported that feeding jejunostomy delayed rather than prevented weight loss; thereby, negating the nutritional benefit of intraoperative feeding jejunostomy [7, 8]. This study aims to evaluate the necessity of feeding jejunostomy during the Ivor Lewis operation by looking at the insertion duration of feeding jejunostomy, prescription of enteral feeding at discharge, and the first outpatient department (OPD) visit and complication rates of feeding jejunostomy.

## Methods

**Fig. 1:**
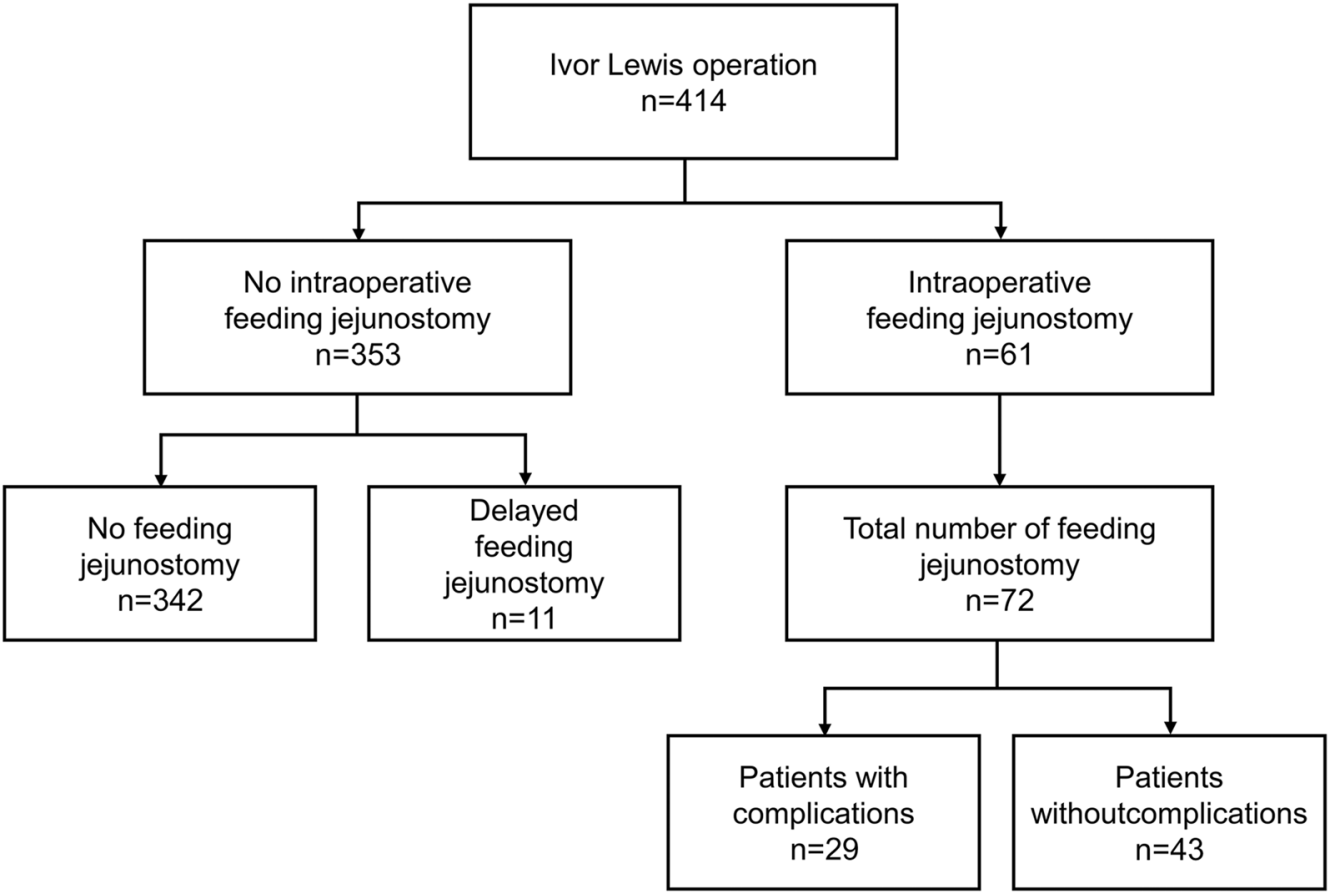
Flow diagram

This was a single-center retrospective study including all esophageal cancer patients who underwent the Ivor Lewis operation between 2015 and 2018. Total of 414 patients underwent the Ivor Lewis operation during the period, and charts were closely reviewed for feeding jejunostomy utility. Figure 1 shows the flow diagram for patients included for the study. Patients were grouped into two categories: intraoperative and no intraoperative feeding jejunostomy groups.

The surgical strategies of the center include open laparotomy with stomach preparation and abdominal lymphadenectomy followed by thoracic lymphadenectomy, transthoracic esophagectomy, and esophageal reconstruction using circular stapler via posterolateral thoracotomy. Most surgeons preferred pyloric drainage procedure (pyloromyotomy or pylomyectomy), and feeding jejunostomy was inserted based on the decision of the surgeon. Indication for feeding jejunostomy insertion includes neoadjuvant CCRT, old age (> 75 years old), malnutrition (body mass index (BMI), < 18.5), poor performance status or poor pulmonary function test (PFT), advanced stage, concurrent stomach cancer, neck dissection or exposure to neck radiotherapy (RT), and mandatory postoperative oral medication.

Feeding jejunostomy was made at 50 cm below the Treitz ligament with 16-Fr. Foley catheter. Two 3–0 black silk were used to form inner and outer purse strings, and a hole was made using electrocautery. The Foley catheter was inserted via the left periumbilical incision. Consequently, purse strings were tied and fixed to the abdominal wall with 3–0 black silk.

Routine postoperative schedule after the Ivor Lewis operations with feeding jejunostomy insertion begins with clamping of feeding jejunostomy and administration of dextrose water at postoperative days (POD) 2 and 3, respectively.

Esophagogastroduodenoscopy (EGD) or esophagography is done at POD 4 or 5, and oral feeding begins after confirmation by the surgeon. Enteral feeding via jejunostomy may be administered as a supportive measure if oral feeding is not sufficient. Most patients omitted the use of feeding jejunostomy if EGD or esophagography showed no sign of anastomosis leakage or graft necrosis because the schedule of oral intake and enteral feeding via jejunostomy coincided. Patients with insufficient oral intake were prescribed enteral feeding at discharge. Feeding jejunostomy was kept if adjuvant chemotherapy was planned or usually removed at the first OPD.

Statistical analysis was performed using chi-square or Fisher’s exact tests for categorical variables. Student *t* or Mann–Whitney *U* tests were used for continuous data. All statistical analyses were performed using the Statistical Package for the Social Sciences, version 24.0.

## Results

This study analyzed 414 consecutive patients who received the Ivor Lewis operation with a diagnosis of esophageal cancer. Sixty-one (14.7%) patients received feeding jejunostomy during operation and 353 patients (85.3%) received esophagectomy without feeding jejunostomy insertion.

Table 1 shows the basic characteristics of the two groups. No significant differences in baseline characteristics were noted between the two groups except for the pathological T stage, where the intraoperative feeding jejunostomy group included higher percentages of T3 and T4. Also, BMI was significantly higher in the no intraoperative jejunostomy group because the indication for feeding jejunostomy insertion includes neoadjuvant CCRT and poor oral feeding before surgery. Although statistically insignificant, patients who received intraoperative feeding jejunostomy were older than patients who did not receive intraoperative feeding jejunostomy.

**Table 1:** Baseline characteristics

	Intraoperative jejunostomy (n = 61)	No intraoperative jejunostomy (n = 353)	*p-*value
Gender, n (%)			0.566
Female	5 (8)	22 (6)	–
Male	56 (92)	331 (94)	–
Age (years old), mean ± SD	67.0 ± 9.3	65.0 ± 8.8	0.110
Body mass index (kg/m^2^), mean ± SD	21.7 ± 3.3	23.2 ± 3.3	0.001
Tumor histology, n (%)			0.767
Adenocarcinoma	3 (5)	17 (5)	–
Squamous cell carcinoma	57 (93)	324 (92)	–
Other	1 (2)	12 (3)	–
Hypertension, n (%)	28 (46)	153 (43)	0.710
Coronary artery diseases, n (%)			
Myocardial infarction	2 (3)	8 (2)	0.634
Angina pectoralis	3 (5)	23 (6)	0.814
Diabetes, n (%)	11 (18)	62 (18)	0.929
Number of pack-years (first–third quartile), median	29.7 (12–40)	27.2 (10–40)	0.446
Pathological tumor stage^a^, n (%)			0.012
T0	12 (20)	59 (17)	–
Tis	1 (2)	1 (0)	–
T1	11 (18)	153 (43)	–
T2	11 (18)	55 (16)	–
T3	24 (39)	81 (23)	–
T4	2 (3)	4 (1)	–
Pathological nodal stage^a^, n (%)			0.177
N0	25 (41)	191 (54)	–
N1	19 (31)	101 (29)	–
N2	12 (20)	43 (12)	–
N3	5 (8)	18 (5)	–

^a^American Joint Committee on Cancer (AJCC) TNM 7th Stage

**Fig. 2:**
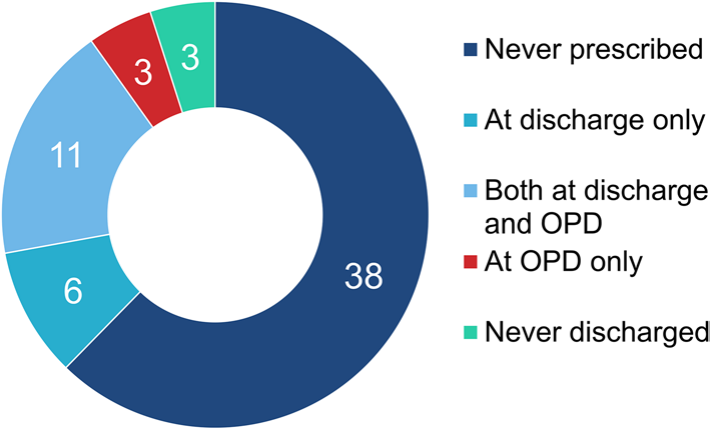
Number of patients with prescription of enteral feeding (Encover^®^)

Table 2 shows the indication for intraoperative feeding jejunostomy. As explained in the Methods section, the indications for feeding jejunostomy insertion were categorized and counted by reviewing the operation record and medical charts. The most common indication for feeding jejunostomy insertion was neoadjuvant CCRT, where 23 patients received intraoperative feeding jejunostomy with the indication. Other indications include old age (> 75 years old), malnutrition (BMI < 18.5), poor PFT or poor performance status, an advanced stage where adjuvant chemotherapy is expected, concurrent stomach cancer, neck dissection or neck radiotherapy, and obligatory requirement for postoperative oral medication. The count exceeds that total number of patients who received intraoperative feeding jejunostomy as some patients had more than one indication for insertion of feeding jejunostomy. Feeding jejunostomy was routinely removed at the first OPD visit, which usually takes place about 4 weeks after the operation. Groups including neoadjuvant CCRT, old age, neck dissection, and obligatory postoperative oral medication had a median duration of feeding jejunostomy insertion less than 30 days which correlated with the first OPD date. Groups including malnutrition and an advanced stage had a relatively longer duration of feeding jejunostomy insertion. Prescription of enteral feeding at discharge or the first OPD clinic visit was also analyzed and showed in Fig. 2. Of the 61 patients who received intraoperative feeding jejunostomy, three patients expired due to postoperative complications and 58 were discharged from the hospital. Eleven (19.0%) patients were prescribed enteral feeding at discharge and at the first outpatient clinic visit. Six patients who were prescribed enteral feeding at discharge did not require further prescription at the first OPD. Three patients who were not prescribed enteral feeding at discharge were prescribed enteral feeding at the first outpatient clinic visit due to poor oral intake. Thirty-eight (65.5%) patients were never prescribed enteral feeding, neither at discharge nor at the outpatient clinics.

**Table 2:** Indication and duration of intraoperative feeding jejunostomy

Indication	n	Average duration (days)	Median duration (days)
Neoadjuvant concurrent chemoradiation therapy	23	34.9	25.5
Old age	14	35.8	25
Malnutrition (body mass index < 18.5)	13	55.2	45
Poor pulmonary function test/poor performance status	12	45.1	34
Advanced stage	11	55.5	50
Stomach cancer	6	64	59.5
Neck dissection/neck radiotherapy	3	33.7	19
Mandatory postoperative medication	3	20.7	22
Number of indication(s)	n	Kept, average (days)	Kept, average (days)
1	36	34.9	25.5
2	25	49.8	38

Among the 353 patients who did not receive intraoperative feeding jejunostomy, 11 patients received delayed feeding jejunostomy insertion. Table 3 shows the indication and duration for delayed feeding jejunostomy insertion. Anastomosis leakage and cancer progression were the most common causes for delayed feeding jejunostomy insertion. Graft failure, acute lung injury, postoperative fistula, and swallowing difficulty were other reasons for delayed feeding jejunostomy insertion. The average duration of feeding jejunostomy insertion was relatively longer for patients with delayed feeding jejunostomy. Two patients with cancer recurrence expired during the follow-up, and the average feeding jejunostomy duration was not counted.

**Table 3:** Indication and duration of delayed jejunostomy insertion

Indication	n = 11	Average duration (days)
Anastomosis leakage	4	59.8
Graft failure	1	45
Cancer recurred	1 (3)	59
Acute lung injury	1	27
Postoperative fistula	1	59
Swallowing difficulty	1	51

**Fig. 3:**
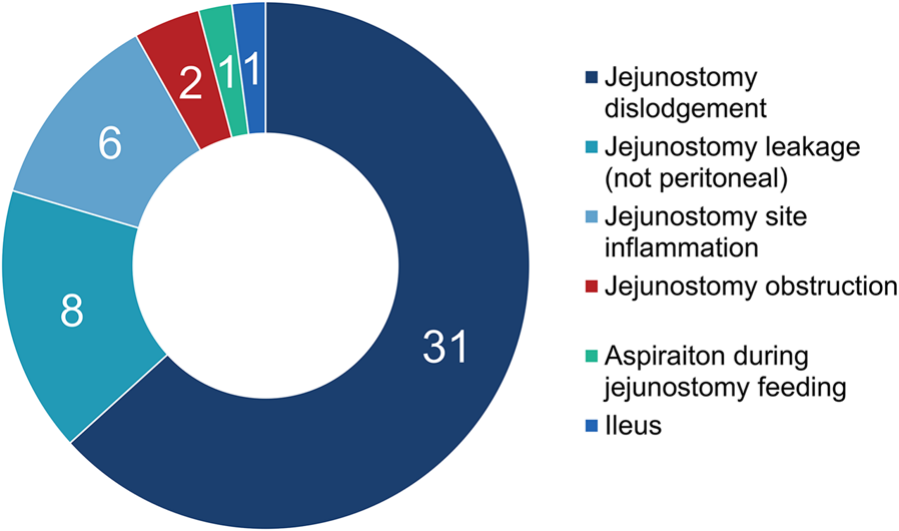
Number of complications related feeding jejunostomy (intraoperative and delayed)

Figure 3 shows the number and distribution of complications related to feeding jejunostomy. There were 29 patients who had complications related to feeding jejunostomy. Many patients had more than one complication, and 41 feeding jejunostomy-related complications were noted. The number of emergency room (ER) visits, unscheduled OPD visits, and readmission due to feeding jejunostomy-related complications were 25, 2, and 1, respectively. There was one patient who had to visit ER four times due to feeding jejunostomy-related complications. Most complications were minor, with jejunostomy dislodgement as the most common complication. In addition, jejunostomy leakage (eight cases), jejunostomy site inflammation (six cases), jejunostomy obstruction (two cases), and aspiration pneumonia after jejunostomy feeding (one patient) were noted. One patient had jejunostomy site-related ileus, which required small bowel resection.

## Discussion

This study demonstrates a low utilization rate (34.5%) of feeding jejunostomy and a relatively high rate (40.8%) of feeding jejunostomy-related complications after the Ivor Lewis operation. Although many investigators have reported that leaks are more common after cervical anastomosis compared to intrathoracic, numerous reports state that cervical anastomosis is equally effective as thoracic anastomosis [9–11]. During the four years (2015–2018), anastomosis leakage occurred at a rate of 3.6%, which correlates to 15 of 414 patients. Only 1 of the 61 patients with intraoperative feeding jejunostomy had anastomosis leakage with postoperative acute respiratory disease syndrome which resulted in an anastomosis fistula with use of steroid. This patient expired during admission. The results show that the anastomosis rate was low after the Ivor Lewis operation, and the results do not vary much when compared to other studies [5, 12, 13]. Moreover, most studies failed to identify predisposing factors for anastomosis leakage [14, 15]. Numerous indications for insertion of feeding jejunostomy with neoadjuvant CCRT, old age, advanced stage, malnutrition, poor PFT, concurrent stomach cancer, neck dissection or RT, and obligatory postoperative oral medication have been adapted. However, an anastomosis leakage rate of 1.6% for selected patients who received intraoperative feeding jejunostomy in this study have shown the difficulty in predicting anastomosis leakage after esophagectomy.

Enhanced recovery after surgery (ERAS) programs are now more widely applied to many fields of surgery, and early oral feeding is one of the key components of the ERAS program. Although early oral feeding is now the standard protocol for colorectal surgeries (on the day or day after surgery), little evidence exists after esophagectomy [6, 16, 17]. The current protocol was similar to the classical protocol with the initiation of oral intake on POD 4–5 after EGD or Esophagography. The duration of nil-by-mouth was shortened over time which, in turn, reduced the need for feeding jejunostomy in the center of this study. Furthermore, Lassen et al. showed the feasibility of early oral intake without increasing postoperative morbidity in upper gastrointestinal surgery. Yet, only eight cases of esophagectomy were included in this study [18]. The recently concluded international, multicenter, and randomized controlled NUTRIETN II trial, conducted to find the effect of the direct start of oral feeding following minimally invasive esophagectomy (MIE) compared with the standard care, has shown that direct oral feeding after an esophagectomy did not increase incidence or severity of postoperative complications [19]. However, the high incidence rate of anastomosis leakage for both the intervention and control groups leaves a question mark. Further studies are needed for more evidence of early oral feeding after esophagectomy and early oral feeding may diminish the need for feeding jejunostomy even more.

The nutritional benefit of feeding jejunostomy is also debatable. The nutritional benefit of enteral feeding has been widely recognized with rare serious complications, with some studies showing the positive effect of feeding jejunostomy on weight loss after esophagectomy and chemotherapy [20–23]. However, there are recent studies which have contradictory results. Carrol et al. [7] compared the change in relative weight and body mass over 1 year between two groups with and without feeding jejunostomy after esophagectomy and reported that routine jejunostomy delays rather than prevent weight loss after esophagectomy. Kroese et al. [24] compared weight loss between the two groups with and without feeding jejunostomy after MIE and claimed that no significant difference in weight loss exists between the groups at 3 and 6 months postoperatively. Koterazawa et al. [25] also showed similar results regarding weight loss after esophagectomy and stated that feeding jejunostomy would increase postoperative complications without improving postoperative malnutrition. The current study did not assess body weight or nutritional status simply because the feeding jejunostomy was rarely used for most patients. Groups with advanced stage and malnutrition had a substantially longer duration of feeding jejunostomy insertion compared to other groups. Many advanced stage patients had adjuvant chemotherapy, and feeding jejunostomy tubes were kept for in cases of poor oral intake during chemotherapy. Subsequently, feeding jejunostomy was removed after chemotherapy was done. Longer insertion duration did not guarantee actual usage of the feeding jejunostomy in this group as these patients were rarely prescribed enteral feeding at discharge nor outpatient clinics. It is also noticeable that patients with two or more indications for insertion of feeding jejunostomy had longer duration of jejunostomy insertion compared to patients with a single indication.

Feeding jejunostomy-related complications were common but not fatal. Previous studies reported that the overall and major complication rates of a surgically placed jejunostomy were 13–38% and 0–3%, respectively [26]. The data showed similar results as 29 (40%) of 72 patients had complications related to feeding jejunostomy. Some patients had more than one complication, with one patient visiting the ER four times with feeding jejunostomy-related complications. 27 patients (38%) had minor complications (e.g., jejunostomy dislodgement, jejunostomy leakage, jejunostomy site inflammation, and jejunostomy site obstruction). Two patients (2.7%) had major complications (one patient had aspiration pneumonia during jejunostomy feeding, and one patient had jejunostomy site related ileus, which required operation). Although minor complications did not change the course of the disease, the quality of life ought to have affected by the complications. Two-thirds of the patients with intraoperative feeding jejunostomy were never prescribed with enteral feeding. Thus, the practicality of intraoperative feeding jejunostomy during the Ivor Lewis operation is being questioned. Moreover, only 11 (3.1%) of 353 patients received delayed feeding jejunostomy. Other 342 patients (96.9%) never needed feeding jejunostomy, although some could get help because 10 patients with anastomosis leakage did not receive delayed feeding jejunostomy.

A limitation of this study is that it is a single-center retrospective study with the possibility of selection bias.

## Conclusions

This study showed that a low rate of feeding jejunostomy usage and a relatively high rate of complications. Feeding jejunostomy was kept for longer durations for advanced stage and malnourished patients for supportive purposes, although patients who were prescribed enteral feeding at discharge did not necessarily correlate. This study suggests that feeding jejunostomy should be reserved only for the exceptionally selected cases with multiple high-risk factors in patients who undergoing Ivor Lewis operation.

## Data Availability

Available.

## Abbreviations

CCRT: Concurrent chemoradiation therapy
OPD: Outpatient department
BMI: Body mass index
PFT: Pulmonary function test
RT: Radiotherapy
POD: Postoperative days
EGD: Esophagogastroduodenoscopy
ER: Emergency room
ERAS: Enhanced recovery after surgery
MIE: Minimally invasive esophagectomy
